# A model of prevention of mother-to-child transmission and health management team for improving adverse outcomes of pregnancy syphilis in Ningxia, China

**DOI:** 10.1186/s12879-024-10029-4

**Published:** 2024-10-10

**Authors:** Chenglei Zhang, Jinwei Yue, Liying Ji, Yongxiang Huang, Qingmei Shi, Xiulian Yang, Jingjiao Wang

**Affiliations:** 1 Medical Laboratory, General Hospital of Ningxia Medical University, Yinchuan, Ningxia, 750003 China; 2 Department of laboratory, Yinchuan women and children healthcare hospital, Yinchuan, Ningxia, 750001 China; 3 Department of Periodontics, Stomatological Hospital, General Hospital of Ningxia Medical University, 804 Shengli South Street, Xingqing District, Yinchuan, 750003 Ningxia China

**Keywords:** Syphilis, Prevention of mother-to-child transmission, Health management, Adverse outcomes, Ningxia

## Abstract

Regional variations exist in the implementation of Syphilis Mother-to-Child Transmission Prevention (PMTCT). Thus, it is crucial to assess the effectiveness of this model in the Ningxia region and explore the supplementary role of Health Management Teams (HMT). This study established the PMTCT + HMT model and examined its impact on adverse outcomes in pregnant women with syphilis infection. The majority of participants were urban residents, married, had a minimum high school education, and held public positions; 36.7% and 26.7% were from minority ethnic groups. The PMTCT + HMT model enhanced participants’ knowledge, rates of voluntary counseling, and testing. The incidence of adverse pregnancy outcomes (miscarriages, preterm births, stillbirths) significantly decreased, and adverse neonatal outcomes (low birth weight, neonatal mortality, congenital syphilis) were notably reduced. Simultaneously, we identified factors associated with adverse outcomes, including non-residency, unmarried status, lower educational attainment, minority ethnicity, primary syphilis, and positive titers. Thus, HMT may be an effective intervention to enhance the effect of PMTCT for syphilis. The unique population structure in Ningxia is closely linked to adverse outcomes, highlighting the significance of providing equitable treatment for vulnerable populations.

## Introduction

Syphilis is a sexually transmitted disease with potential transmission from mother to child, and its impact should not be underestimated. Pregnant women with syphilis may encounter adverse outcomes, including miscarriage, stillbirth, and congenital syphilis in newborns, posing a significant threat to maternal and infant safety. Global statistics indicate that approximately 2 million pregnant women contract syphilis annually, with a high risk of mother-to-child transmission (MTCT) ranging from 60 to 80%. It is estimated that around 25% of these cases may result in the mentioned adverse outcomes [1, 2]. Syphilis is also considered a crucial indicator for assessing the public health status of countries or regions. Its occurrence and transmission show distinct geographical clustering, mainly in low- and middle-income countries and regions. This phenomenon may be linked to insufficient public health systems and incomplete prevention of mother-to-child transmission [3, 4]. Therefore, improving the efficiency of public health systems and enhancing prevention and control measures for MTCT are crucial to reduce syphilis spread and protect maternal and infant health.

In China, the prevalence of syphilis among pregnant women is around 0.24% [5]. Alarmingly, about 14% of syphilis infections lead to severe adverse outcomes [6], imposing a substantial burden on both society and maternal and child health. Since the initiation of the World Health Organization’s (WHO) Prevention of Mother-to-Child Transmission (PMTCT) program, China has prioritized interrupting syphilis mother-to-child transmission, leading to the development of the China Syphilis Prevention and Control Plan (2010–2020) [7]. However, there are still systemic and lagging issues in interrupting syphilis mother-to-child transmission in certain regions, especially in less developed provinces such as Ningxia Hui Autonomous Region, situated in the inland northwest. In these areas, where development is relatively slower, the implementation of the PMTCT model to prevent and intervene in syphilis mother-to-child transmission is still imperfect.

Therefore, this study aims to expand the PMTCT model within a specified scope, leveraging the strengths of healthcare institutions. It introduces Health Management Teams (HMT) to enhance health education, deliver personalized interventions, and implement monitoring measures, addressing potential gaps in PMTCT model application. The research objective is to validate the effectiveness of the PMTCT + HMT model in preventing mother-to-child transmission of syphilis and examine its association with sociodemographic characteristics of the local population. This study aims to establish a theoretical foundation for developing precise strategies to interrupt syphilis mother-to-child transmission, thereby reducing the societal burden, and improving maternal-child health.

## Subjects and methods

### Subjects

A total of 150 pregnant women who underwent prenatal examinations and were diagnosed with syphilis at the General Hospital of Ningxia Medical University and Yinchuan Maternal and Child Health Care Hospital from January 2020 to December 2022 were selected as the study subjects. The patients were randomly divided into the intervention group (90) and the control group (60) by using completely simple randomized and single-blind methods. This study was approved by the Medical Research Ethics Committee of General Hospital of Ningxia Medical University (Protocol No.: 2018 − 367), and informed consent was obtained from all subjects (Clinical trial number: MR-64-24-034238).

### Methods

#### Establishment of the PMTCT/HMT model

In the context of PMTCT, the fundamental strategy is centered around “priority on prevention, effective monitoring, early diagnosis, and standardized treatment.” Building upon this foundation, a Health Management Team (HMT) is formed, consisting of 5 doctors and 6 laboratory technicians, covering clinical medicine, reproductive medicine, and preventive healthcare. Prior to intervening with the research subjects, the team members undergo focused training on the pathogenesis, transmission routes, prevention, and treatment measures associated with syphilis. They then tailor health management procedures, content, and methods according to the specific circumstances, with the aim of enhancing the theoretical knowledge, educational skills, and managerial capabilities of the team members.

#### Intervention programs

The intervention plan strictly adheres to the “Expert Consensus on Diagnosis and Management of Syphilis Complicating Pregnancy“ [7]. It includes the following components: (a) Prenatal Screening and Diagnosis: All pregnant women undergo syphilis serological screening during their initial prenatal examination following conception. This screening encompasses quantitative syphilis testing, Treponema pallidum particle agglutination assay (TPPA), and rapid plasma reagin (RPR) syphilis testing. It is recommended to initiate the first prenatal examination within the first three months of pregnancy, given the prevalence of latent syphilis in pregnant women. Emphasis is placed on serological screening. (b) Treatment Principles and Regimens: Early and standardized treatment is imperative. Treatment with benzathine penicillin is administered in varying dosages and durations, contingent upon the disease’s progression, staging, and the presence of complications. For individuals with drug allergies, the decision to switch to alternative treatment is contingent on the efficacy of desensitization therapy. (c) Follow-up: Serological testing is ideally performed on a monthly basis to assess changes in the condition and the effectiveness of treatment, thereby determining the need for additional intervention. In the case of newborns, the decision to administer medication is based on the mother’s treatment during pregnancy and the serological examination of the newborn. Building upon this foundation, the intervention group also implemented a health management team member responsibility system. This system aims to enhance health education for pregnant women by emphasizing the risks associated with syphilis and mother-to-child transmission. It includes the provision of testing and treatment plans. Additionally, it encourages the partners of pregnant women to undergo syphilis testing. Simultaneously, comprehensive supervision and management are carried out throughout the process. This involves monitoring changes in key indicators and adhering to standardized treatment protocols. Moreover, essential psychological interventions are provided as part of the holistic approach.

#### Observation indicators

The health knowledge/awareness, rate of voluntary counseling, number of tests, satisfaction, as well as adverse pregnancy outcomes and adverse neonatal outcomes were compared between the two groups. A questionnaire consisting of 20 questions was designed to assess the mastery of health knowledge, including general prevention knowledge such as “routes of syphilis transmission” and “syphilis testing for sexual partners,” as well as professional questions such as “staging and classification of syphilis” and “clinical manifestations of syphilis at different stages.” The voluntary consultation rate was calculated based on the number of participants who actively sought consultation or communication. Satisfaction was measured using a self-designed questionnaire that objectively evaluated research projects and the research team, including items such as “support and guidance received during the study” and “assessment of the research team’s professionalism and communication skills.”

### Statistical analysis

Measurement data are expressed as mean ± standard deviation, and count data are presented in numbers and percentages. The two groups were compared using Student’s *t* test, and inter-group comparisons were based on Chi-square test. Odds ratios (OR) and 95% confidence intervals (CI) were used to assess factors associated with adverse outcomes. All the analyses were completed using the SPSS 18.0 software package, and a *P* value of<0.05 was considered statistically significant.

## Results

### Data of subjects

The demographic information revealed no significant differences between the two groups. The mean age was 29.58 years in the control group (*n* = 60) and 28.34 years in the intervention group (*n* = 90). The majority of subjects in both groups were locals (61.7% in the control group vs. 73.3% in the intervention group), married (86.7% vs. 92.2%), had secondary school or higher education (83.3% vs. 86.7%), and were employed in public sectors (53.3% vs. 51.1%). Ethnic minorities accounted for 36.7% and 26.7% in the control and intervention groups, respectively. Most pregnant women had a history of syphilis (96.7% vs. 98.9%) and received relevant treatments (88.3% vs. 78.9%). The first syphilis test showed mostly negative RPR titers (76.7% vs. 81.1%), with only 5.0% and 1.2% of cases having RPR titers ≥ 1:4. No subjects exhibited secondary or higher-stage syphilis (all *P* > 0.05) (Table 1).

**Table 1:** Demographic characteristics and syphilis-related information of the subjects in the two groups (*n* = 150)

	Control group (*n* = 60)	Intervention group (*n* = 90)	t/χ2	*P* value
Age (yrs)	29.58 ± 5.73	28.34 ± 5.50	1.329	0.186
Residency				
Locals	37 (61.7)	66 (73.3)	2.277	0.131
Non-locals	23 (38.3)	24 (26.7)		
Occupation				
Public employees	32 (53.3)	46 (51.1)	0.133	0.936
Business persons	17 (28.3)	28 (31.1)		
Others	11 (18.3)	16 (17.8)		
Education background				
Primary school	8 (13.3)	11 (12.2)	0.971	0.808
Secondary school	41 (68.3)	64 (71.1)		
College or above	9 (15.0)	14 (15.6)		
Unknown	2 (3.3)	1 (1.1)		
Ethnicity				
Han	38 (63.3)	66 (73.3)	1.693	0.193
Hui	22 (36.7)	24 (26.7)		
Marital status				
Married	52 (86.7)	83 (92.2)	1.235	0.267
Unmarried	8 (13.3)	7 (7.8)		
Previous history of syphilis	58 (96.7)	89 (98.9)	0.907	0.341
History of previous treatments	53 (88.3)	71 (78.9)	2.241	0.134
Stage of syphilis				
Latent	46 (76.7)	73 (81.1)	0.434	0.510
Primary	14 (23.3)	17 (18.9)		
RPR titer				
Negative	48 (80.0)	76 (84.4)	2.333	0.506
1:1	7 (11.7)	9 (10.0)		
1:2	2 (3.3)	4 (4.4)		
≥ 1:4	3 (5.0)	1 (1.2)		

### Compliance behaviors

The study examined the subjects’ understanding of syphilis and mother-to-child transmission (MTCT), satisfaction levels, and variations in voluntary counseling rates and test frequencies between the two groups. A self-designed questionnaire was employed for this investigation. Within the control group, merely 17 pregnant women (28.3%) possessed adequate knowledge/awareness, while 22 subjects (36.7%) demonstrated insufficient understanding. The lowest answer rate focused on the clinical manifestations and treatment of syphilis. Furthermore, the rate of voluntary counseling in this group was 36.7%. Consequently, the percentage of subjects undergoing ≥ 3 syphilis tests during pregnancy was merely 31.7%, accompanied by an dissatisfaction rate of 11.7%. In contrast, the intervention group exhibited a noteworthy enhancement in knowledge/awareness acquisition. The rate of adequate knowledge/awareness rose to 47.8%, while the rate of inadequate knowledge/awareness decreased to 17.8%. The intervention group witnessed an increase in the rate of voluntary counseling (58.9%), and 61.1% of pregnant women underwent ≥ 3 syphilis tests. The dissatisfaction rate during pregnancy decreased significantly to only 2.2% (All *P* < 0.05) (Table 2).

**Table 2:** Compliance behaviors of subjects in the two groups (*n* = 150)

	Control group (*n* = 60)	Intervention group (*n* = 90)	t/χ2	*P* value
Knowledge/awareness				
Good	17 (28.3)	43 (47.8)	8.476	< 0.05
Average	21 (35.0)	31 (34.4)		
Poor	22 (36.7)	16 (17.8)		
Voluntary counseling	22 (36.7)	53 (58.9)	7.111	< 0.05
Number of tests for syphilis				
≥ 3	19 (31.7)	55 (61.1)	23.393	< 0.001
2	24 (40.0)	32 (35.6)		
1	17 (28.3)	3 (3.3)		
Satisfaction				
Satisfied	23 (38.3)	62 (68.9)	15.586	< 0.001
Somewhat satisfied	30 (50.0)	26 (28.9)		
Dissatisfied	7 (11.7)	2 (2.2)		

### Adverse outcomes

In the control group of 60 subjects, the occurrence of adverse pregnancy outcomes was 25.0%, encompassing 8 cases of miscarriage, 5 cases of preterm labor, and 2 cases of stillbirth. Additionally, the incidence of adverse neonatal outcomes was 26.0%, involving 5 cases of low-birth-weight babies, 2 cases of neonatal death, and 6 cases of congenital syphilis. Conversely, in the intervention group of 90 subjects, the rate of adverse pregnancy outcomes was 3.3%, comprising 1 case of miscarriage and 2 cases of preterm labor. Furthermore, the incidence of adverse neonatal outcomes was 4.5%, involving 3 cases of low-birth-weight babies and 1 case of congenital syphilis, with no recorded neonatal deaths, demonstrating a significant decrease (Table 3).

**Table 3:** Adverse pregnancy and neonatal outcomes in the two groups (*n* = 150)

	Control group (*n* = 60)	Intervention group (*n* = 90)	χ2	*P* value
Adverse pregnancy outcomes				
Miscarriage	8 (13.3)	1 (1.1)	16.761	< 0.001
Preterm labor	5 (8.3)	2 (2.2)		
Stillbirth	2 (3.3)	0 (0.0)		
Adverse neonatal outcomes				
Low-birth-weight babies	5 (10.0)	3 (3.4)	17.052	< 0.001
Neonatal death	2 (4.0)	0 (0.0)		
Congenital syphilis	6 (12.0)	1 (1.1)		

### Factors associated with adverse outcomes

In Table 4, we observed significantly lower probabilities of adverse pregnancy outcomes and neonatal issues among local residents (OR = 0.21; OR = 0.24). Conversely, individuals with a higher educational background (OR = 0.09) and those employed in the public sector (OR = 0.45) exhibited a reduced likelihood of adverse outcomes. Notably, ethnic minority pregnant women had a higher likelihood (OR = 4.12), which was substantially reduced in the intervention group (OR = 1.11). Furthermore, unmarried individuals had a higher probability of adverse outcomes (OR = 10.33), a probability that significantly decreased following the interventions (OR = 6.24). Additionally, the stage of syphilis and the RPR titer exhibited positive correlations with adverse outcomes. Primary syphilis (OR = 3.89) and a positive RPR titer (OR = 8.33) were identified as risk factors for adverse outcomes.

**Table 4:** Factors associated with adverse outcomes (*n* = 150)

	Categorical variables		Control group (*n* = 60)		Intervention group (*n* = 90)	
			OR (95% CI)	*P* value	OR (95% CI)	*P* value
Residency	Locals	Non-locals	0.21 (0.07–0.61)	< 0.05	0.24 (0.06–0.97)	0.057
Occupation	Public employees	Business persons and others	0.45 (0.16–1.33)	0.128	0.70 (0.17–2.74)	0.649
Education background	College and higher	Primary school and secondary school	0.09 (0.01–0.59)	< 0.05	0.88 (0.07–6.59)	< 0.913
Ethnicity	Hui	Han	4.12 (1.42–12.22)	< 0.05	1.11 (0.21–5.89)	< 0.906
Marital status	Unmarried	Married	10.33 (1.55–119.50)	< 0.05	6.24 (1.01–32.01)	< 0.05
Stage of syphilis	Primary	Latent	3.89 (1.09–12.33)	< 0.05	7.18 (1.71–30.14)	< 0.05
RPR titer	Positive	Negative	8.33 (1.79–40.03)	< 0.05	9.73 (2.24–41.32)	< 0.05

## Discussion

Mother-to-child transmission (MTCT) is a primary route for the transmission of syphilis. The disease can be transmitted vertically through the transplacental route, during delivery, or through breastfeeding, resulting in adverse outcomes such as miscarriage, stillbirth, or neonatal syphilis. MTCT often occurs due to low coverage and detection rates of syphilis testing during pregnancy. To meet the WHO goal of globally eliminating MTCT of syphilis, China integrated a Prevention of Mother-to-Child Transmission (PMTCT) program for syphilis into its maternal and child healthcare efforts in 2010. The program was implemented by adapting PMTCT programs for HIV/AIDS. This adaptation contributed to the annual increase in maternal syphilis seroprevalence (from 2.03‰ in 2011 to 3.05‰ in 2018), closely linked to the continuous expansion of syphilis screening coverage for pregnant women [8]. Despite a significant increase in the treatment workload, the incidence of neonatal syphilis declined from 0.92‰ to 0.18‰ [9]. Although the PMTCT model has been initially confirmed as effective, it has shortcomings, including geographical disparities due to varying socio-economic levels and imperfections in healthcare systems that cannot be neglected.

As a national autonomous region with an ethnic minority population of nearly 36%, Ningxia faces challenges such as a lower socio-economic level and limited healthcare capacity. Consequently, the implementation of the PMTCT model in this region remains incomplete. In this study, we introduced the HMT approach, modeled after the health management program in Liberia [10]. This approach involved medical staff from various institutions and disciplines. Importantly, our approach was implemented on a smaller yet more focused scale. The distinct division of labor, strategic deployment, streamlined workflow, and standardized processes enhanced the cohesion of closed-loop management, ensuring the effectiveness of the PMTCT model. Effective health education markedly enhanced the knowledge and awareness of pregnant women, leading to a reduction in the proportion of women with inadequate knowledge/awareness from 36.7 to 17.8%. As a result, the rate of voluntary counseling increased, and the doctor-patient relationship improved through enhanced communication. This led to adequate syphilis tests during pregnancy, contributing to a substantial increase in maternal satisfaction. Furthermore, the expertise and teamwork among HMT members were further enhanced.

Our results were conclusive and promising. The PMTCT/HMT model significantly decreased adverse pregnancy outcomes when applied throughout gestation. The incidence of stillbirth decreased from 3.3 to 0%, consistent with earlier studies [11, 12]. Additionally, there was a notable reduction in the risks of miscarriage and preterm birth. Moreover, it proved particularly effective in preventing congenital syphilis and led to more favorable outcomes in neonates. As suggested by the WHO, the effectiveness of PMTCT for syphilis is influenced by various factors, such as management patterns and timely, effective antenatal care [13]. Our current study also revealed lower incidence rates of unfavorable outcomes among local residents, potentially linked to the higher rates of early diagnosis and treatment within this population. Similarly, *Zhang et al.* proposed that environmental conditions, like proximity to residence and traffic conditions, were factors influencing maternal access to prenatal care [14]. Business individuals, those with a low educational background, and unmarried individuals were deemed more susceptible to adverse outcomes. Hence, more equitable models and policies should be devised for these vulnerable groups. Additionally, ethnic differences demonstrated certain influences on adverse outcomes, possibly associated with the unique religion and culture of the Hui ethnic group in this autonomous region. Finally, both the primary stage of syphilis and a positive RPR titer were identified as risk factors for adverse outcomes, consistent with numerous prior studies [15, 16]. It was also noted that high RPR titers (1:8 and above) in pregnant women posed a greater risk. Therefore, the detection and reduction of serological titers are crucial.

Our study has limitations. Firstly, we validated the PMTCT model and explored the HMT in a small geographical area with a limited sample size, potentially affecting the accuracy of the results. Secondly, due to participant and environmental constraints, our study included only pregnant women with syphilis. Consequently, we were unable to analyze adverse outcomes in pregnant women without syphilis, resulting in a lack of control group data in larger samples. Lastly, our present study did not fully investigate maternal and fetal factors as well as external reasons, which could contribute to adverse outcomes. Despite these limitations, our study is the first to verify the PMTCT model of syphilis in Ningxia, potentially offering valuable insights for syphilis prevention in this region. Future studies will expand sample sizes and collect comprehensive information on study subjects to better evaluate the effectiveness of PMTCT of syphilis in Ningxia.

## Conclusion

HMT may be an effective intervention to enhance the effect of PMTCT for syphilis. The unique demographic structure in Ningxia is closely associated with unfavorable outcomes. Efforts should focus on promoting equitable treatment for the disease among vulnerable populations. Additionally, attention should be given to both the stage of syphilis and positive RPR titers as risk factors for adverse outcomes.

## Data Availability

The data that support the findings of this study are available from the corresponding author upon reasonable request.
